# *Kalanchoe tubiflora* extract inhibits cell proliferation by affecting the mitotic apparatus

**DOI:** 10.1186/1472-6882-12-149

**Published:** 2012-09-10

**Authors:** Yi-Jen Hsieh, Ming-Yeh Yang, Yann-Lii Leu, Chinpiao Chen, Chin-Fung Wan, Meng-Ya Chang, Chih-Jui Chang

**Affiliations:** 1 Department of Laboratory Medicine and Biotechnology, Tzu Chi University, Hualien, 97004, Taiwan; 2 Department of Molecular Biology and Human Genetics, Tzu Chi University, No. 701, Zhongyang Rd., Sec. 3, Hualien, 97004, Taiwan; 3 Institute of Medical Sciences, Tzu Chi University, Hualien, Taiwan; 4 Graduate Institute of Natural Products, Chang Gung University, Taoyuan, Taiwan; 5 Department of Chemistry, National Dong-Hwa University, Hualien, Taiwan; 6 School of Applied Chemistry, Chung Shan Medical University, No.110,Sec.1,Jianguo N.Road, Taichung City, 40201, Taiwan; 7 Institute of NanoEngineering and MicroSystems, National Tsing Hua University, No. 101, Section 2, Kuang-Fu Road, Hsinchu, 30013, Taiwan; 8 Department of Medical Research, Buddhist Tzu-Chi General Hospital, Hualien, Taiwan

**Keywords:** *Kalanchoe tubiflora*, Multipolar spindle, Anti-proliferation

## Abstract

**Background:**

*Kalanchoe tubiflora* (KT) is a succulent plant native to Madagascar, and is commonly used as a medicinal agent in Southern Brazil. The underlying mechanisms of tumor suppression are largely unexplored.

**Methods:**

Cell viability and wound-healing were analyzed by MTT assay and scratch assay respectively. Cell cycle profiles were analyzed by FACS. Mitotic defects were analyzed by indirect immunofluoresence images.

**Results:**

An n-Butanol-soluble fraction of KT (KT-NB) was able to inhibit cell proliferation. After a 48 h treatment with 6.75 μg/ml of KT, the cell viability was less than 50% of controls, and was further reduced to less than 10% at higher concentrations. KT-NB also induced an accumulation of cells in the G2/M phase of the cell cycle as well as an increased level of cells in the subG1 phase. Instead of disrupting the microtubule network of interphase cells, KT-NB reduced cell viability by inducing multipolar spindles and defects in chromosome alignment. KT-NB inhibits cell proliferation and reduces cell viability by two mechanisms that are exclusively involved with cell division: first by inducing multipolarity; second by disrupting chromosome alignment during metaphase.

**Conclusion:**

KT-NB reduced cell viability by exclusively affecting formation of the proper structure of the mitotic apparatus. This is the main idea of the new generation of anti-mitotic agents. All together, KT-NB has sufficient potential to warrant further investigation as a potential new anticancer agent candidate.

## Background

The mitotic spindle is a complex molecular scaffold that mediates proper sister-chromatid segregation during mitosis and is essential in maintaining genomic integrity of daughter cells. In animal cells, thus far two major pathways have been identified in mitotic spindle assembly. First, the mitotic centrosomes function as microtubule-organizing centers (MTOCs) [1], directing spindle assembly from spindle poles through the regulation of nucleating microtubules [2]. The second pathway is dependent on a RanGTP gradient mediated by Aurora A, which regulates spindle assembly [3,4]. The chromosomal passenger complex (CPC) generates spindle assembly in the absence of a RanGTP gradient [5,6]. Disruption of these pathways and disruption of the mitotic spindle will activate the spindle assembly checkpoint, causing mitotic arrest and cell death [4].

Compounds that target microtubule assembly are often very promising as anti-cancer medication. Vinca alkaloids and taxanes, for example, are administered against both solid tumors and haematological malignancies [7-9]. Many of these compounds were originally isolated from a variety of marine organisms or botanicals [10-12]. Microtubule-targeting drugs are classified into two groups, microtubule-destabilizing agents and microtubule stabilizing agents [13,14]. Both microtubule -destabilizing and -stabilizing agents inhibit cell proliferation by binding to tubulin, altering microtubule dynamics, and disrupting the mitotic spindle. Cancer cells are generally more susceptible to tubulin binding agents compared to normal cells [15]. Since microtubules are present in cells that are in interphase or undergoing mitosis, these microtubule targeting agents not only disrupt mitotic spindle formation but they can also disrupt the microtubule network. The microtubule network mediates important cellular processes during interphase, which include cellular migration and intracellular transport. Disruption of the microtubule network can cause a diverse range cytotoxic effects [16].

Recently, a new generation of anti-mitotic agents that target kinesins and mitotic kinases, such as Eg5, Aurora kinase and polo-like kinase, were developed as putative anti-cancer medications [17,18]. Eg5 is a kinesin motor that localizes to the centrosomes and MT asters during prophase [19] and is required for centrosome separation as well as bipolar spindle assembly [20,21]. The aurora kinase family is crucial for the progression of cells from mitosis to cytokinesis [22-25]. Aurora A localizes to spindle poles and functions in centrosome maturation, separation and spindle bipolarity [26,27]. Aurora B is the enzymatically active member of the CPC, which localizes along the chromosome arms and at the centromeres during prophase. It is concentrated at the inner centromere region from prometaphase to metaphase and transfers to the spindle midzone, cell cortex and midbody during late mitosis and cytokinesis [25,28]. The CPC is essential both for the assembly and stability of the bipolar mitotic spindle [29]. Inhibition of Aurora B and other components of CPC causes mitotic catastrophe [29-32], indicating that CPC may be involved in both mitotic spindle assembly pathways.

*Kalanchoe* is a genus of the Family Crassulaceae. Various species of *Kalanchoe* are often referenced in folklore, and commonly used in traditional medicine worldwide for the treatment of fever, abscesses, bruises, contused wounds, coughs, skin diseases, infections, hypertension, rheumatism and inflammation [33-36]. *Kalanchoe* species are also used by the Kerala tribes for treating cancer symptoms [37]. A variety of bufadienolide compounds were isolated from various *Kalanchoe* species, which show strong anti-tumor promoting activity [38-43]. *Kalanchoe tubiflora* (KT), due to its wide variety of potential biological activities, was selected for this study. KT is one of the most common medicinal plants used for wound healing in Southern Brazil. The traditional uses of KT in wound healing coincide with results from systematic biological assays [44]. Here we show that an n-BuOH-soluble fraction of KT has anti-proliferative activity, which is due to the induction of multi-polar spindles and chromosomal misalignment of mitotic cells. These abnormal mitotic events lead to mitotic catastrophe, a desirable effect of a cancer therapeutic drug.

## Methods

### Regents

All reagents were purchased from Sigma unless otherwise stated. The primary antibodies were used as followed: anti-alpha tubulin (mouse mAb B512, 1:2000; Sigma, Taiwan); anti-Aurora A polyclonal antibody (1: 500, Cell signaling, Taiwan); anti-phospho-Histone 3 polyclonal antibody (1: 500, Upstate, Taiwan).

### Preparation of extracts from Kalanchoe tubiflora

**Figure 1:**
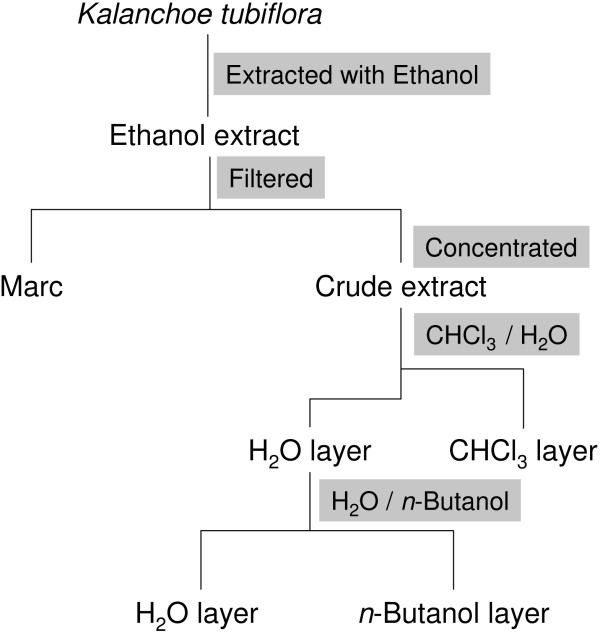
**Flow chart of *Kalanchoe tubiflora *extract synthesis.**

Fresh *Kalanchoe tubiflora (KT)* was chopped and boiled three times with 95% EtOH under reflux and filtered. The filtered broth was concentrated under reduced pressure. The crude extract was resuspended in H_2_O and partitioned successively with CHCl_3_ and n-BuOH to give a CHCl_3_ -soluble fraction (KT-C), a n-BuOH-soluble fraction (KT-NB), and a H_2_O-soluble fraction (KT-W). 53.79 g of dry (KT-NB) extract were obtained from 6638.76 g of raw KT plant tissue. The procedure of KT-NB extraction is represented in figure 1. Stock solution was prepared in DMSO and filtered through 0.22 μm membrane. Cell culture medium was used to further dilute the extracts to a desired concentration for all cellular assays.

### Cell lines and culture

Human lung carcinoma (A-549), human bladder papillary transitional cell carcinoma (BFTC905), human breast carcinoma (MCF-7), human larynx Epidermoid carcinoma (HEp-2), human uterus sarcoma (MED-SA) and HeLa cells were purchased from the Food Industry Research and Development Institute (Hsinchu, Taiwan). Cells were grown, according to supplier instructions, in media supplemented with 10% fetal bovine serum (FBS). Cells were maintained at 37°C in a humidified atmosphere of 5% CO2.

### Cell viability assay

The percentage of growth inhibition was determined by the MTT cell proliferation/viability assay. A total of 2000 cells/well were seeded onto a 96-well plate for 16 h. Cells were treated with various concentrations of KT-NB (25 μg/ml, 12.5 μg/ml, 6.25 μg/ml, 3.125 μg/ml, and 1.5625 μg/ml) and incubated for an additional 3 days at 37°C. The MTT assay was performed in triplicate. Subsequently, the test solutions were removed and replaced with culture medium containing 500 μg/ml of MTT (Thiazolyl blue formazan, Sigma, Taiwan) for an additional 4-6 h. The supernatant was aspirated, and 200 μl of DMSO was added to the wells to dissolve any precipitate present. The optical density (OD) values were measured in an ELISA reader (SUNRISE, TECAN, Switzerland) at a wavelength of 570 nm. The mean and standard deviation of each group were calculated. The OD reading of every group was first subtracted by a blank (background) control. Relative survival rate = [OD (treatment groups)/OD (negative control)] x 100.

### Wound-healing assay

Cell mobility was assessed using a wound-healing assay. A-549 cells were seeded in six-well plates for 24 h. Confluent monolayer cells were scratched by a 200 μl pipette tip and then removed by washing the cells with serum-free medium to clear cell debris and suspension cells. Migration of cells into the wound was observed at different time points. Cells that migrated into the wounded area or cells with extended protrusion from the border of the wound were visualized and photographed under an inverted microscope.

### FACS

Cells were treated with KT-NB or DMSO and harvested at 24, 48, 72 h. Floating and adherent cells were collected and fixed in cold 70% ethanol at 4°C overnight. After washing, cells were treated with RNase and stained with Propidium Iodide (PI) for 1 h in the dark at room temperature. Flow cytometric analysis was performed by using a FACScalibur flow cytometer (Becton Dickinson). Cell cycle distribution was analyzed using Cell Quest software (Becton Dickinson). Each experiment was conducted three times.

### Immunofluorescence and microscopy

For immunostaining, cells were fixed in 4% paraformaldehyde in PBS buffer for 10 min at room temperature. The cells were permeabilized in 0.1% Triton X-100 in PBS for 5 min and then rinsed in PBS. Cells were blocked for 30 min at room temperature in PBS + 10% FBS. Antibody incubations were performed in PBS for 1 h, followed by four 10-min washes in PBS at room temperature. DNA was stained with 0.1 mg/ml DAPI for 5 min at room temperature and rinsed with PBS. Slides were mounted in Vectashield mounting medium (Vectra) and sealed using nail varnish.

Images were performed using a Zeiss Axiovert 200 M microscope controlled by MetaMorph Software. Image stacks were deconvolved and quick-projected. The images were processed using Adobe Photoshop.

## Results

### KT-NB decreases cell viability

**Figure 2:**
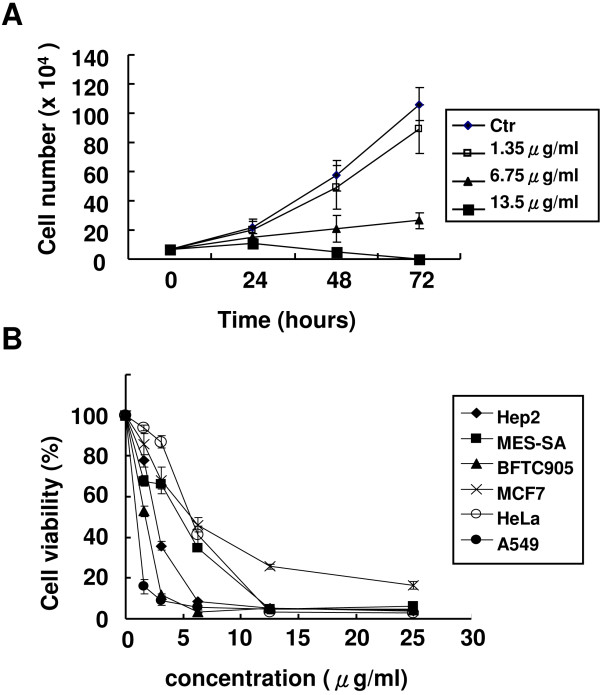
**KT-NB inhibits cell proliferation and reduces cell viability.** (**A**) HeLa cells were treated with different concentration of KT-NB (1.35 μg/ml, 6.75 μg/ml, 13.5 μg/ml). DMSO was used as control. Cell numbers were determined using a haemocytometer at different time points. (**B**) Human lung carcinoma (A-549), human bladder papillary transitional cell carcinoma (BFTC905), human breast carcinoma (MCF-7), human larynx Epidermoid carcinoma (HEp-2), human uterus sarcoma (MED-SA) and HeLa cells were treated with 1.5625 μg/ml, 3.125 μg/ml, 6.25 μg/ml, 12.5 μg/ml, 25 μg/ml of KT-NB for 72 h and harvested for MTT assay. Results were based on three independent experiments.

We treated HeLa cells with different doses of KT-NB to analyze its effect of cell proliferation. Cells were exposed to three different concentrations of KT-NB and DMSO, which was used as a control. At each time point, the cell number was determined using a haemocytometer. Compared to control cells, 1.35 μg/ml of KT-NB only slightly affected cell growth; however, cell growth was inhibited at concentrations of 6.75 μg/ml and 13.5 μg/ml after a 48 h treatment (Figure 2A). We also used the MTT assays to assess the anti-proliferative effect of KT-NB on Hep2, MES-SA, BFTC905, MCF7, HeLa and A549 cells (Figure 2B). These cells were treated with KT-NB (1.5625 μg/ml, 3.125 μg/ml, 6.25 μg/ml, 12.5 μg/ml, and 25 μg/ml) for 72 h and harvested for analysis. KT-NB reduced the viability of the different cancer cells in a dose dependant manner. The IC50 of KT-NB in A549 cells was less than 1.5625 μg/ml. KT-NB could also inhibit the growth of other cancer cells at slightly higher concentrations (6.25 μg/ml). Compared to cancer cells, the toxicity of KT-NB was less in normal cells (Additional file 1: Figure S1). It is worthy of note that there was ambiguity whether the reduced cell growth rate resulted from cell death or non-proliferating. Overall, these results showed that KT-NB could effectively inhibit the growth of cancer cells at concentrations less than 6.25 μg/ml.

### KT-NB inhibits cell migration

**Figure 3:**
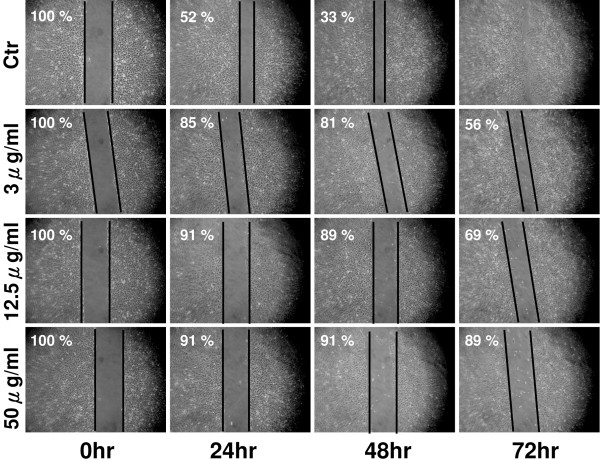
**KT-NB inhibits cell migration in a wound healing assay.** A-549 cells were seeded in six-well plates and allowed to adhere for 24 h. Cells were treated with 3 μg/ml, 12.5 μg/ml, 50 μg/ml of KT-NB. Migration of cells into the wound was observed at 24, 48, 72 h after monolayer cells were scratched by a 200 μl pipette tip. Results are based on three independent experiments. Lines indicated the border of the wounds. Imagines were processed using Photoshop software. The contrast of the images was adjusted to that the border could be identified without ambiguity. The border lines were determined by eyes.

A wound healing assay was performed to assess the effect of KT-NB on A549 cell migration. In response to a scratch wound, cells treated with KT-NB (3 μg/ml, 12.5 μg/ml, and 50 μg/ml) exhibited a concentration and time dependent decrease in cell migration. Cell migration was slightly inhibited at lower KT-NB concentrations (3 μg/ml), while at the concentration of 50 μg/ml, KT-NB almost completely blocked the migration of cells into the scratch wound (Figure 3). Note that this inhibition effect might due to either inhibition of cell migration or induction of cell death after KT-NB treatment.

### KT-NB induces cell cycle arrest

**Figure 4:**
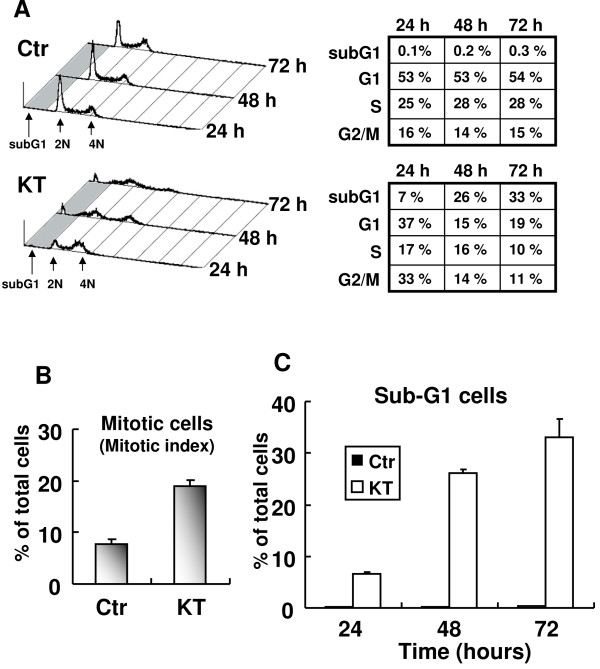
**KT-NB arrests cells in the M phase and increases sub-G1 cell population.** (**A**) FACS analysis of DNA content after KT-NB treatment. HeLa cells were treated with 6.75 μg/ml of KT-NB or DMSO as control. Cells were harvested for analysis after 24, 48 and 72-h treatment. Tables showed the percentage of relative cell numbers of different cell cycle phases. (**B**) Cells were treated with 6.75 μg/ml KT-NB for 24 h. Quantitation of the mitotic index in control and KT-NB treated cells (n > 500, three independent experiments). (**C**) Percentage of relative cell numbers of sub-G1 cells. Cells were calculated based on the results shown in (**A**).

FACS analysis was performed to examine the effect of KT-NB on the cell cycle. After 24 h treatment of KT-NB, a progressive accumulation of cells were found in the G2/M phase of the cell cycle, which was accompanied with a decrease of cells in the G1 phase (Figure 4A). The mitotic index in control and KT-NB treated cells was examined by both anti-phospho-Histone H3 staining and DAPI staining under a fluorescence microscope. The mitotic index increased from 8% in control cells to 20% in cells treated with 6.75 μg/ml of KT-NB at 24 h (Figure 4B). We also observed that cells exposed to KT exhibited higher levels of subG1 with DNA content less than 2 N. The level of cells in the subG1 phase increased to 35% after 72 h treatment (Figure 4C).

### KT-NB induces multipolar spindles

**Figure 5:**
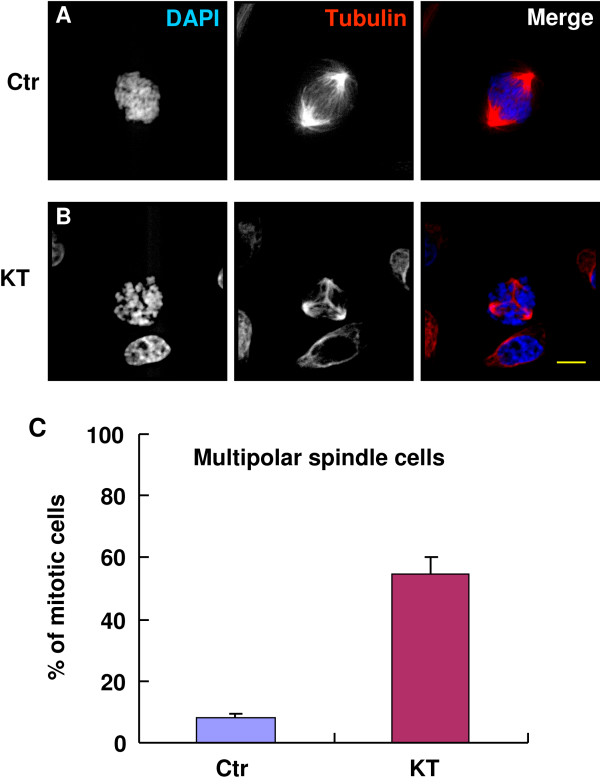
**KT-NB induces multipolar spindles.** HeLa cells were treated with DMSO as a control (**A**) or 6.75 μg/ml of KT-NB (**B**) for 24 h. After incubation, cells were fixed and processed for immunofluorescence analysis. DAPI was used for DNA staining, while anti-tubulin shows the mitotic spindle. Merged images: (DAPI is blue, tubulin is red). (**C**) The percentage of mitotic cells with multipolar spindles was quantified in control and KT-NB treated cells (n > 100, three independent experiments). Scale bar: 5 μm.

**Figure 6:**
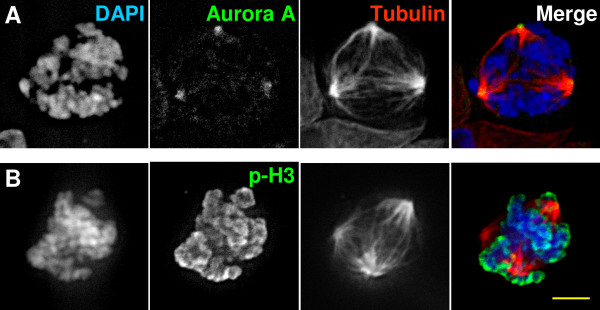
**KT-NB does not affect Aurora A localization and histone H3 phosphorylation on serine**^**10**^**.** HeLa cells were treated with 6.75 μg/ml of KT-NB for 24 h and harvested for immunofluorescence analysis. DAPI was used for DNA staining, while anti-tubulin shows the mitotic spindle. Cells were immunostained with. anti-Aurora A (**A**) or Anti-phospho-histone H3 (Ser10) (**B**). Merged images DAPI is blue, anti-tubulin is red, Aurora A (**A**) or histone H3 (**B**) is green. Scale bar: 5 μm.

Since KT-NB treatment induces cell cycle arrest during mitosis, we analyzed whether KT-NB could cause mitotic spindle defects and regulate important mitotic checkpoints. To analyze the structure of mitotic spindles, we used HeLa cells to performed immunofluorescent staining. The mitotic phases of cells were carefully analyzed by DAPI and microtubule staining. Majority of the control cells showed perfect bipolar structure during metaphase (Figure 5A). In contrast, cells treated with 6.75 μg/ml of KT-NB for 24 h exhibited multipolar spindles (Figures 5B, 6A and 6B). More than half of the mitotic cells had more than two spindle poles (Figure 5C). To examine whether KT-NB treatment can alter important components involved in the mitotic spindle poles, the localization of Aurora A was analyzed. Cells treated with KT-NB showed normal Aurora A localization to spindle poles (Figure 6A).

Aurora B is required for stability of the bipolar mitotic spindle [32] and functions through the phosphorylation of histone H3 on Ser10 and Ser28 in prophase. Our results indicated that KT-NB treated cells also did not exhibit altered levels of Histone H3 phosphorylation (Figure 6B). Immunofluorescence analysis of α-tubulin and γ-tubulin revealed that the microtubule organization and centrosome number were normal in cells treated with KT-NB in interphase (data not shown). Taken together, these results suggested that KT-NB treatment induced multipolar spindles but did not interfere with the recruitment of essential components necessary for functional spindle poles, nor did it disrupt the microtubule network in interphase cells.

### KT-NB induces chromosome misalignment in metaphase

**Figure 7:**
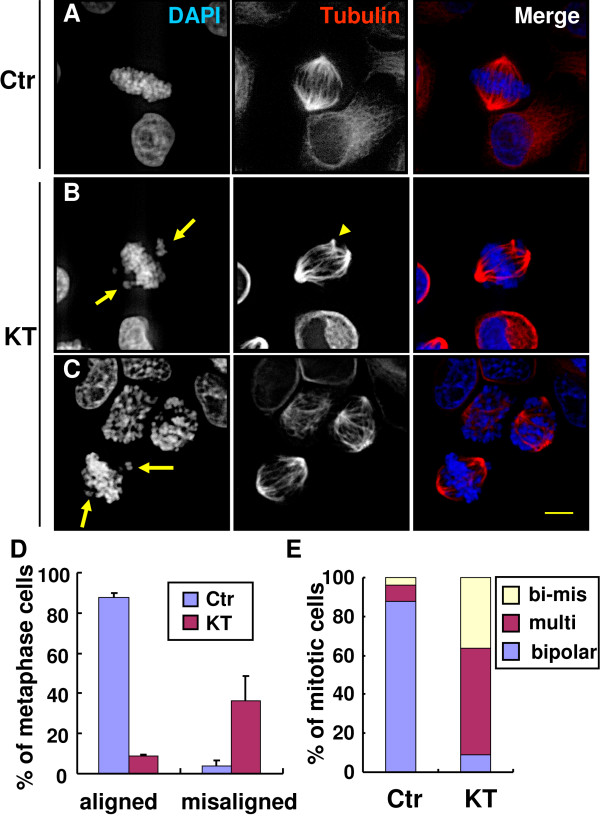
**KT-NB induces chromosomal misalignment in cells with bipolar spindles.** Cells were treated with DMSO as control (**A**) or 6.75 μg/ml of KT-NB (**B**, **C**) for 24 h. After incubation, cells were fixed and processed for immunofluorescence analysis. (**A**) The control cell in metaphase showed bipolar spindle with chromosome alignment to the metaphase plate. (**B**) The KT-NB treated cell showed bipolar spindle containing extra centrosome (arrowhead). (**C**) The KT-NB treated cells exhibited bipolar spindle with the normal two centrosomes. Arrows points to the misalignment chromosomes. DAPI was used for DNA staining, while anti-tubulin shows the mitotic spindle. Merged images: (DAPI is blue, tubulin is red). Scale bar: 5 μm. (**D**) Only the metaphase cells were analyzed. The percentage of metaphase cells with misaligned chromosome(s) in the bipolar spindle were quantified in control and KT-NB treated cells (n > 50, three independent experiments). (**E**) All phases of mitotic cells were analyzed. The percentage of mitotic cells with multipolar spindles or misaligned chromosome(s) was quantified in control and KT-NB treated cells (n > 100, three independent experiments). Bi-mis: cells with bipolar spindle and misaligned chromosomes. Multi: cells with multipolar spindles.

KT-NB treatment induced a large amount of mitotic cells with multipolar spindles, but 45% of the mitotic cells still showed normal bipolar spindles. Unexpectedly, KT-NB-treated cells with normal bipolar spindle assembly experienced problems in the chromosomal congression to the metaphase plate (Figures 7B and 7C). To specify this phenotype, we only analyzed metaphase cells. In cells treated with KT-NB for 24 h, 37% of the metaphase cells with bipolar spindles contained misaligned chromosomes (Figure 7D). In contrast, control cells in metaphase exhibited correct chromosomal alignment at the metaphase plate (Figure 7A). It is worthy to note that high percentage of mitotic cells with mitotic apparatus defects was observed if all phases of mitotic cells were analyzed. By grouping cells that exhibited misaligned chromosomes with bipolar spindles or that exhibited multipolar spindles (i.e. abnormal mitotic cells), the level of defective mitotic cells after KT-NB treatment was, at 90%, much higher than control cells (Figure 7E).

## Discussion

Medicinal plants are an important source for the potential development of effective anticancer agents [45]. In fact, more than half of the today’s anticancer drugs were originally synthesized from natural products and their derivatives. In the present study, we found that extracts from *Kalanchoe tubiflora* (KT), exhibited significant anti-proliferative effects against a variety of human cancer cell lines.

Majority of the effective anti-cancer or antibiotic concentrations of plant extracts are greater than 100 μg/ml [45,46]; however, in our study, cell proliferation was inhibited by 48 h treatment with 6.75 μg/ml KT-NB, and the viability was less than 50% after a 72 h exposure. KT-NB does affect normal diploid human cells that are dividing but much less efficiently than most of the cancerous cell lines we tested. Therefore, KT-NB may be a highly effective candidate as a future anti-cancer drug.

Drugs based on natural products that bind to tubulin or microtubules remain an important component in chemotherapy. Anti-mitotic agents inhibit cell proliferation by suppressing microtubule dynamics [13]; however, based on immunofluorescence staining images, we found that KT-NB did not disrupt the microtubule organization in interphase or spindle formation during mitosis, indicating that it is not a microtubule destabilizing agent. [47]. Our results also showed that KT-NB, which did not lead to the formation of parallel microtubule alignment or packed bundles of microtubules, is not a microtubule stabilizing agent either [48]. Rather than acting on microtubule dynamics, we found that KT-NB induces multipolar spindles and perturbs accurate mitosis.

Centrosomes increase both in size and in microtubule-capacity in late G2 phase of the cell cycle. Aurora A, by recruiting pericentriolar material (PCM) and interacting with the Ran-TPX2 pathway, is required for the maturation of centrosomes and mitotic-spindle assembly respectively [3,49]. It was noted that depletion of TPX2 from Xenopus egg extracts results in the formation of less compact spindles [50]. In vertebrate cells, depletion of TPX2 using RNAi also caused the formation of multipolar spindles [51]; however, inhibition of Aurora A activity results in monopolar spindles [24]. Our immunostaining results showed that localization of Aurora A was not disrupted by KT-NB. Since we did not observe a high level monopolar spindles, an effect common with Aurora A inhibitors, it is also unlikely that KT-NB inhibits Aurora A activity.

Some of the KT-NB-treated cells showed normal bipolar spindles with two centrosomes in mitosis. It was noted that a few cells with more than two centrosomes could assemble almost bipolar spindles (Figure 7B). With KT-NB treatment, it did not matter whether spindles were formed by the normal two centrosomes or by extra centrosomes, as more than half of the cells with bipolar spindles failed to exhibit properly aligned chromosomes at the metaphase plate. Although, chromosomal misalignment can result from the suppression of microtubule dynamics by a stabilizing poison, such as taxol [48], as discussed previously, this is an unlikely mechanism for KT-NB.

The failure of non-bipolar attachment corrections during the stochastic attachment process in prometaphase, is another possibility for chromosomal misalignment. Aurora B, the enzymatic member of CPC, destabilizes the non-bipolar microtubule-kinetochore attachments by phosphorylating several microtubule capture factors on the kinetochore [52-54]. To examine if the misaligned chromosome in KT-NB treatment was due to the inhibition of Aurora B kinase, we analyzed the histone H3 phosphorylation levels. In prophase, the CPC localizes to the chromosome arms where it phosphorylates histone H3 on Ser10 and Ser28 [25]. Phosphorylation of histone H3 was not affected in KT-NB treated cells (Figure 6B).

Plk is a central regulator of the cell cycle, playing crucial roles in mitotic events, including activation of CDC25c phosphatase, regulation of microtubule nucleation, centrosome maturation and kinetochore assembly [55,56]. To ensure the accuracy of these processes, plk1 activity is subjected to complex regulation. It is activated at mitotic entry by Aurora A kinase and its adapator BORA, which phosphorylate plk1 in its T-loop [57,58]. Plk1 is also activated by Aurora B kinase at centromeres, and that is ccrucial for polo function in regulating chromosome dynamics in prometaphase [59]. In our study, monopolar spindles were not observed in KT-NB treated cells. Two other possible targets of KT-NB are CENP-E and Mps1. CENP-E is a plus end-directed motor protein that has a pivotal role in mitosis. It stabilizes the interaction between microtubules and kinetochores of the mitotic spindle [60] and regulates the mitotic checkpoints by modulating the function of BuBR1 [61]. Complete inhibition of CENP-E leads to defective mitosis with unaligned chromosomes and apoptosis [62]. Mps1 was originally discovered in a yeast genetic screen for mutants producing monopolar spindle [63]. It is a conserved multi-functional kinase that plays roles in the SAC and chromosome bi-orientation [64]. Some small-molecular inhibitors of Mps1 were developed in 2010 [65]. Chemical inhibition of Mps1 leads to chromosome misalignment and increases the frequency of multipolar mitoses [66].

David Pellman’s group used live cell images to study the fate of cells with extra centrosomes in 2009 [67]. According to their study, most of progeny of multipolar cells died or arrested regardless of whether the cells were mono- or poly-nucleated. It will be interesting to use live cell images in the future to reveal the fate of KT-NB treated cells.

Targeting of mitotic cells is one of the bases of therapy for patients with multiple types of solid tumors. Some antimitotic agents, taxanes or vinca alkaloids, affect both dividing and nondividing cells. An essential characteristic of the ideal new generation of antimitotic agents is to target dividing cells but not non-dividing cells. As most cancer cells have faster dividing rates, these drugs target cancer cells preferentially. Potentially then, perturbing the mitotic apparatus by KT-NB may be used to more efficiently target rapidly proliferating cancer cells.

## Conclusions

Here we report that KT-NB is a promising anti-cancer agent candidate. Further *in vivo* research will be needed to determine the effectiveness of KT-NB as an actual anti-cancer compound. Unlike microtubule binding agents, KT-NB inhibits cell proliferation and reduces cell viability by two mechanisms: first, by inducing multipolarity; second, by disrupting chromosome alignment during metaphase. Both mechanisms disrupt mitotic progression and inhibiting cell proliferation. The underlying mechanism of KT-NB is complicated and unclear. Further research involving the identification and isolation of pure compounds that parallel the phenotypes observed in KT-NB treated cells will be necessary to determine the cellular targets of KT-NB.

## Competing interests

The authors declare that there are no conflicts of interest.

## Authors’ contributions

YJH carried out extraction, wound healing assay, data analysis. MYY carried out immunostaining, MTT assay. YLL collected plant and identified *Kalanchoe tubiflora.* CPC and CFW participated in extraction design. MYC carried out microscopy. CJC conceived of the study, and participated in its design and coordination and helped to draft the manuscript. All authors read and approved the final manuscript.

## Pre-publication history

The pre-publication history for this paper can be accessed here:

http://www.biomedcentral.com/1472-6882/12/149/prepub

## Supplementary Material

Additional file 1 **Figure S1.** The toxicity of KT-NB was less in normal cells. (A) Normal female embryonic lung cells (WI-38) and lung cancer cells (A549) were treated with different concentrations of KT-NB (1.35 μg/ml, 6.75 μg/ml, 13.5 μg/ml and 25 μg/ml). DMSO was used as a control. Cells were harvested for MTT assay at different time points. The absorbance of the control group was defined as 100%. Results were based on three independent experiments. (B) Cell morphology of WI-38 was unaffected after KT-NB treatment for 72 h. Click here for file
