# Supplementary figures and images for: Kalanchoe tubiflora extract inhibits cell proliferation by affecting the mitotic apparatus
Source: BMC Complement Altern Med. 2012 Sep 10;12:149. doi: 10.1186/1472-6882-12-149 (PMC3557174; doi:10.1186/1472-6882-12-149)

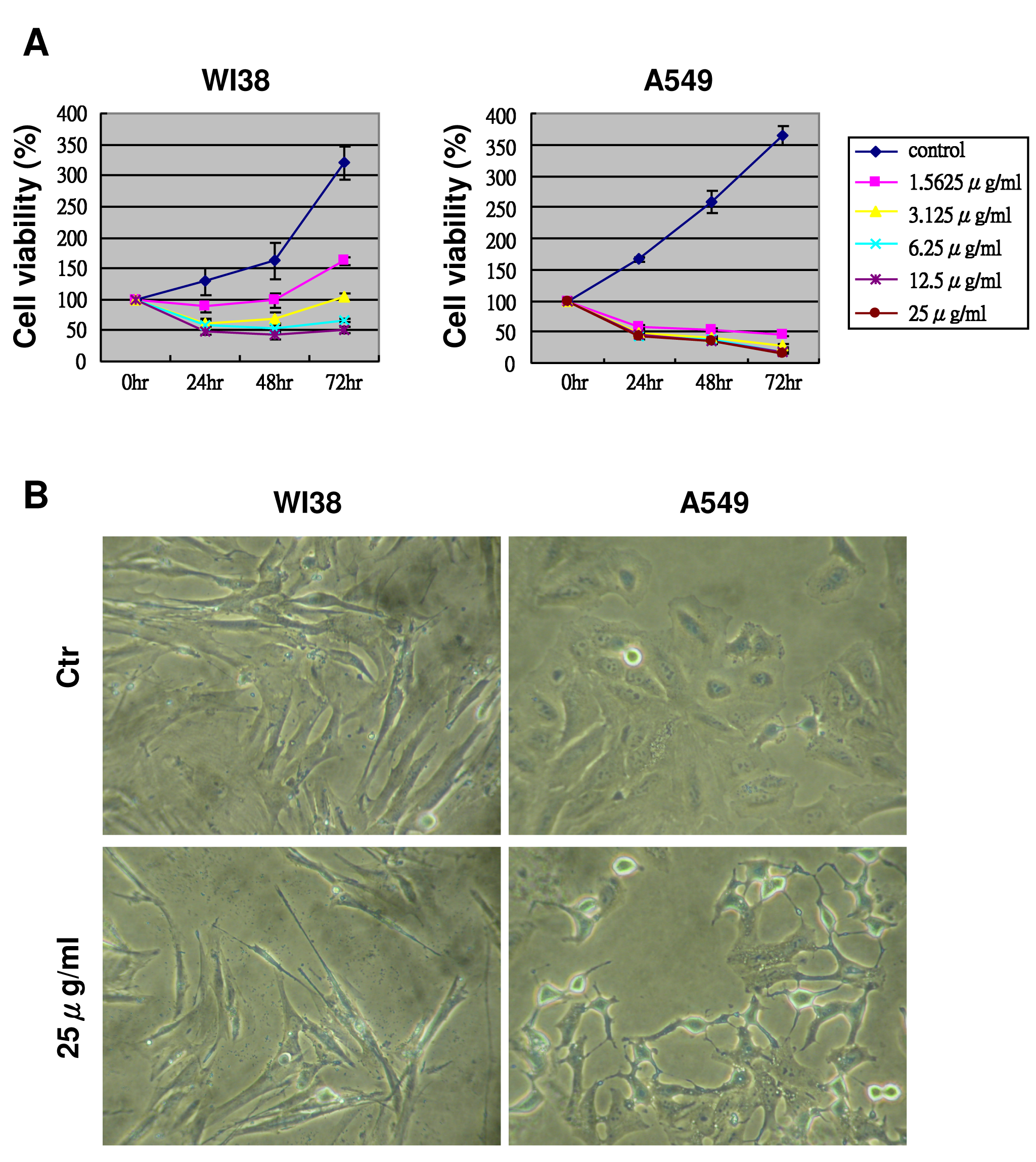

Supplement: Additional file 1 — Figure S1. The toxicity of KT-NB was less in normal cells. (A) Normal female embryonic lung cells (WI-38) and lung cancer cells (A549) were treated with different concentrations of KT-NB (1.35 μg/ml, 6.75 μg/ml, 13.5 μg/ml and 25 μg/ml). DMSO was used as a control. Cells were harvested for MTT assay at different time points. The absorbance of the control group was defined as 100%. Results were based on three independent experiments. (B) Cell morphology of WI-38 was unaffected after KT-NB treatment for 72 h. [file 1472-6882-12-149-S1.tiff]
